# Striatal responsiveness to reward under threat‐of‐shock and working memory load: A preliminary study

**DOI:** 10.1002/brb3.1397

**Published:** 2019-09-26

**Authors:** Claudie Gaillard, Matthias Guillod, Monique Ernst, Salvatore Torrisi, Andrea Federspiel, Dominik Schoebi, Romina E. Recabarren, Xinyi Ouyang, Christoph Mueller‐Pfeiffer, Antje Horsch, Philipp Homan, Roland Wiest, Gregor Hasler, Chantal Martin‐Soelch

**Affiliations:** ^1^ IReach Lab Unit of Clinical and Health Psychology Department of Psychology University of Fribourg Fribourg Switzerland; ^2^ Section on Neurobiology of Fear and Anxiety National Institute of Mental Health Bethesda MD; ^3^ Psychiatric Neuroimaging Unit Translational Research Center University Hospital of Psychiatry and Psychotherapy University of Bern Bern Switzerland; ^4^ Unit of Clinical Family Psychology Department of Psychology University of Fribourg Fribourg Switzerland; ^5^ iBM Lab Department of Psychology University of Fribourg Fribourg Switzerland; ^6^ Department of Consultation‐Liaison‐Psychiatry and Psychosomatic Medicine University Hospital Zurich University of Zurich Zurich Switzerland; ^7^ Department Woman‐Mother‐Child Lausanne University Hospital Lausanne Switzerland; ^8^ Institute of Higher Education and Research in Healthcare University of Lausanne Lausanne Switzerland; ^9^ Center for Psychiatric Neuroscience Feinstein Institute for Medical Research New York NY; ^10^ Department of Diagnostic and Interventional Neuroradiology University Hospital of Bern Bern Switzerland; ^11^ Unit of Psychiatry Research University of Fribourg Fribourg Switzerland

**Keywords:** anticipation, delivery, fMRI, reward, stress, striatum, working memory

## Abstract

**Introduction:**

Reward and stress are important determinants of motivated behaviors. Striatal regions play a crucial role in both motivation and hedonic processes. So far, little is known on how cognitive effort interacts with stress to modulate reward processes. This study examines how cognitive effort (load) interacts with an unpredictable acute stressor (threat‐of‐shock) to modulate motivational and hedonic processes in healthy adults.

**Materials and Methods:**

A reward task, involving stress with unpredictable mild electric shocks, was conducted in 23 healthy adults aged 20–37 (mean age: 24.7 ± 0.9; 14 females) during functional magnetic resonance imaging (fMRI). Manipulation included the use of (a) monetary reward for reinforcement, (b) threat‐of‐shock as the stressor, and (c) a spatial working memory task with two levels of difficulty (low and high load) for cognitive load. Reward‐related activation was investigated in a priori three regions of interest, the nucleus accumbens (NAcc), caudate nucleus, and putamen.

**Results:**

During anticipation, threat‐of‐shock or cognitive load did not affect striatal responsiveness to reward. Anticipated reward increased activation in the ventral and dorsal striatum. During feedback delivery, both threat‐of‐shock and cognitive effort modulated striatal activation. Higher working memory load blunted NAcc responsiveness to reward delivery, while stress strengthened caudate nucleus reactivity regardless reinforcement or load.

**Conclusions:**

These findings provide initial evidence that both stress and cognitive load modulate striatal responsiveness during feedback delivery but not during anticipation in healthy adults. Of clinical importance, sustained stress exposure might go along with dysregulated arousal, increasing therefore the risk for the development of maladaptive incentive‐triggered motivation. This study brings new insight that might help to build a framework to understand common stress‐related disorders, given that these psychiatric disorders involve disturbances of the reward system, cognitive deficits, and abnormal stress reactivity.

## Graphical abstract

Reward and stress are important determinants of motivated behaviors. Of clinical significance, a major challenge is to understand under which circumstances motivated behaviors might get disrupted. This study examines how cognitive effort interacts with an unpredictable acute stress exposure (threat‐of‐shock) to modulate motivational and hedonic processes in healthy adults. Our results provide initial evidence that both stress and cognitive load modulate striatal responsiveness during feedback delivery but not during anticipation in healthy adults. This study brings new insight that might help to build a framework to understand common stress‐related disorders, given that these psychiatric disorders involve disturbances of the reward system, cognitive deficits, and abnormal stress reactivity.

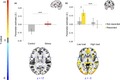

## 1 INTRODUCTION

The ability to detect potential rewards and threats in the environment is fundamental for the survival of humans and animals (Haber & Knutson, 2009). Reward is defined as the positive value that one ascribes to an object, an action, or an internal physical state, and as a value that elicits approach behavior (Schultz, Dayan, & Montague, 1997; Wise, 2004). In contrast, imminent threat stimulates the autonomic nervous system, leading to a “fight‐or‐flight” response to escape or avoid the aversive situation (McEwen, 2007). When a threat persists over time, uncertainty leads to a sustained state of vigilance or avoidance (Bali & Jaggi, 2015; Grillon, 2008). Therefore, adaptive goal‐directed behaviors build on the capacity to attribute a value to both positive and negative stimuli in order to promote approach toward rewards or avoidance of threats (Balleine, Delgado, & Hikosaka, 2007; Fareri & Tottenham, 2016). Although reward‐related approach behaviors and threat‐related defensive responses are mainly mediated by subcortical systems, the ability to control reactions and actions is modulated by cortical regions involved in cognitive processes, especially working memory (Gilbert & Fiez, 2004; LeDoux & Pine, 2016; Pochon et al., 2002).

Research demonstrates the involvement of a corticostriatal circuit in reward processes (Fiallos et al., 2017; Fuentes‐Claramonte et al., 2015; Liu, Hairston, Schrier, & Fan, 2011; Tanaka, Pan, Oguchi, Taylor, & Sakagami, 2015). In particular, the striatum, including its ventral and dorsal subdivisions, plays a crucial role in detecting potential rewards and in modulating consecutive reward‐driven behaviors (Delgado, 2007; Haber & Knutson, 2009). Part of the ventral striatum (Choi, Yeo, & Buckner, 2012), the nucleus accumbens (NAcc) is mainly engaged in affective valuation of positive and negative incentives, contributing to motivated actions such as avoidance or approach behaviors in both animals and humans (for a review see: Balleine & Killcross, 2006; Gottfried, O'Doherty, & Dolan, 2003; Pedroni, Koeneke, Velickaite, & Jäncke, 2011). To date, the role of the ventral striatum in reward anticipation has been widely evidenced both in animals (Ikemoto & Panksepp, 1999) and in humans (Diekhof, Kaps, Falkai, & Gruber, 2012; Knutson, Adams, Fong, & Hommer, 2001; Knutson, Fong, Adams, Varner, & Hommer, 2001; O'Doherty, Deichmann, Critchley, & Dolan, 2002; Rademacher, Salama, Gründer, & Spreckelmeyer, 2013). Its implication has been shown in prediction errors reflecting deviations of received rewards from expected rewards (Hare, O'Doherty, Camerer, Schultz, & Rangel, 2008; Wittmann et al., 2016), and in the encoding and representation of reward value and magnitude (for reviews, see Bartra, McGuire, & Kable, 2013; Diekhof et al., 2012). With respect to the dorsal striatum, the caudate nucleus is implicated in the selection of appropriate goal‐directed actions based on the evaluation of action‐outcome associations, while the putamen governs more automatized behaviors that are restricted to stimulus–response associations (Grahn, Parkinson, & Owen, 2008). In experimental settings, reward processing is often parsed into its motivational and hedonic subcomponents according to two temporal phases, (a) reward anticipation and (b) reward delivery. The former is related to the motivation to obtain a rewarding incentive (i.e., a “wanting” component), whereas the latter represents the hedonic state elicited by the reward delivery (i.e., a “liking” component) (Berridge, 2009; Berridge & Kringelbach, 2013; Berridge, Robinson, & Aldridge, 2009; Luking, Pagliaccio, Luby, & Barch, 2016). Two competing systems called model‐based and model‐free learning are involved in the control of action selection and motivated behaviors (Lee, Shimojo, & O'Doherty, 2014). In model‐based learning, motivated behaviors result from the evaluation of contingencies between an instrumental action and its outcome (e.g., a positive reinforcer or a reward) and in the computation of action value which promote goal‐directed behaviors (Balleine & Dickinson, 1998; Daw, Niv, & Dayan, 2005). While the emergence of goal‐directed behaviors is initiated by model‐based learning, they progressively become more habitual and automatized through a transition to model‐free learning, anchored in a stimulus–response mechanism (Everitt & Robbins, 2013). These two learning processes implicate different neural substrates, with goal‐directed behaviors mainly governed by the NAcc and the caudate nucleus, while habit formation is essentially controlled by the putamen (Burton, Nakamura, & Roesch, 2015; Everitt & Robbins, 2013).

Dysfunctions in reward‐seeking and goal‐oriented behaviors are common symptoms of several prevalent psychiatric conditions, such as addiction (Koob, Gilpin, & Boutrel, 2013; Martin‐Soelch, 2013; Nikolova & Hariri, 2012), major depression (Alloy, Olino, Freed, & Nusslock, 2016), eating disorders (Avena & Bocarsly, 2012; Keating, Tilbrook, Rossell, Enticott, & Fitzgerald, 2012), or schizophrenia (Hanssen et al., 2015; Strauss, Waltz, & Gold, 2014). For instance, depressed patients show perturbations in the brain systems involved in reward valuation and associated approach behaviors, resulting consequently in a loss of motivation, interest, or pleasure for activities, which were previously rewarding (Admon & Pizzagalli, 2015; Hägele et al., 2015; Martin‐Soelch, 2009). In turn, drug dependence is characterized by a decrement in the rewarding effect of nondrug rewards coupled with an amplified incentive salience of cues predicting drug‐related rewards (Koob, 2008, 2010; Koob et al., 2013; Martin‐Soelch, 2013; Martin‐Soelch et al., 2001; T. E. Robinson & Berridge, 2000). According to the opponent process theory (Solomon, 1980), this pathological motivational process develops through the positive hedonic feelings elicited by the drug, which result consequently in the positive reinforcement of drug‐seeking behaviors. Following the positive hedonic effect, a counterregulatory homeostatic mechanism comes into play to restore the body's homeostasis compromised by the overstimulation produced by the drug intake (for a review, see George & Koob, 2010). This negative process is associated with the recruitment of brain stress systems and with the emergence of negative emotional states, which are thought to precipitate drug consumption to relieve the negative consequences of withdrawal (Martin‐Soelch, 2013).

Converging with the role played by the engagement of brain stress systems in pathological motivated behaviors, acute stressors are known to alter both the sensitivity to reward (Berghorst, Bogdan, Frank, & Pizzagalli, 2013; Pizzagalli, Bogdan, Ratner, & Jahn, 2007) and the core executive functions (for a review, see Shields, Sazma, & Yonelinas, 2016), in particular working memory (Oei et al., 2016; Qin, Hermans, van Marle, Luo, & Fernández, 2009; Zandara et al., 2016). Acute stressors are defined as time‐limited threats to an organism (Pacák & Palkovits, 2001). In experimental settings, acute stressors consist of threats lasting one hour or less (Dickerson & Kemeny, 2004), while unpredictable acute stress is known to elicit anxiety and cognitive deficits (Bali & Jaggi, 2015). In turn, chronic stressors refer to sustained or repeated threats over one week or more (Armario, 2015). Brain imaging data revealed that acute, chronic, and early‐life stress exposure altered neural reactivity to reward in animals (Kleen, Sitomer, Killeen, & Conrad, 2006; Lin, Bruijnzeel, Schmidt, & Markou, 2002; Willner, Moreau, Nielsen, Papp, & Sluzewska, 1996) and humans (Berghorst et al., 2013; Boecker et al., 2014; Bogdan & Pizzagalli, 2006; Ginty, 2013; Hanson et al., 2015; Porcelli, Lewis, Delgado, Tobler, & Schwabe, 2012). In humans, experimental acute stressors, such as threat‐of‐shock or the cold pressor test, were found to impair reward‐related neural responses in the ventral striatum during both reward anticipation (Choi, Padmala, Spechler, & Pessoa, 2013) and feedback delivery (Kumar et al., 2014; Porcelli et al., 2012). Accordingly, psychosocial stress induced by the Trier Social Stress Test (TSST; Kirschbaum, Pirke, & Hellhammer, 1993) was shown to reduce reward responsiveness to sexual stimuli during the anticipatory phase (Oei, Both, van Heemst, & van der Grond, 2014). Blunted brain reactivity to reward under stress was supported at the behavioral level, with decreased ability to modulate behavior as a function of reinforcement schedule in individuals with increased perceived stress in daily life (Pizzagalli et al., 2007). These findings indicating a stress‐induced reduction in reward responsiveness offer a promising neurobiological substrate for understanding the development of anhedonic symptoms that are characteristic of stress‐related disorders including major depression and addiction for instance. In contrast, acute stress has been also linked to amplified incentive‐triggered motivation as evidenced by the enhanced striatal responses to reward under social stress, in particular during the anticipation of monetary reward (Kumar et al., 2014) and of primary rewards (i.e., food; Pool, Brosch, Delplanque, & Sander, 2015). This is in line with the hypothesis that under stressful conditions, rewards may be sought for the stress‐reducing capacity associated with their consumption (Berridge & Robinson, 1998; Koob & Le Moal, 2001). Taken together, findings remain inconsistent so far and call for a better understanding of the factors involved in the modulation of stress‐related effects on reward responsiveness during both anticipatory and delivery processes.

The cognitive effort to expend for obtaining the reward is a crucial factor that might modulate the effect of stress on motivational and hedonic processes, both in experimental settings and in everyday life. In daily life, stressful contexts often accompany demanding tasks, requiring high attentional resources. To achieve a better understanding of how stress and cognition interact to modulate the reward processes, it is necessary to determine how each of these factors per se influences motivation and hedonic experience. Previous research has focused on the complex relationship between cognition, motivation, and hedonic capacities (Akaishi & Hayden, 2016; Esterman et al., 2016; O'Connor, Rossiter, Yücel, Lubman, & Hester, 2012; Rothkirch, Schmack, Deserno, Darmohray, & Sterzer, 2014). A large body of research evidenced the effort‐discounting effect on reward valuation, so that the effort exerted to obtain a desired reward decreases as effort cost increases (for a review, see Kurniawan, 2011). Converging with this hypothesis, higher demanding tasks were shown to decrease the activation in the ventral (Botvinick, Huffstetler, & McGuire, 2009; Croxson, Walton, O'Reilly, Behrens, & Rushworth, 2009; Salamone, Correa, Farrar, & Mingote, 2007) and dorsal (Kurniawan et al., 2010) striatum. In turn, evidence suggests that executive functions, and more specifically working memory capacity, play a critical role in motivational and hedonic processes (Yee & Braver, 2018). The working memory, defined as the capacity for temporarily maintaining and manipulating information (Baddeley, 2010; Collette & Van der Linden, 2002), is a particularly relevant cognitive function to investigate because of its broad implications in learning, reasoning, valuating, planning goal‐directed behavior, and regulating adaptively emotions (Collette & Van der Linden, 2002; Etkin, Buechel, & Gross, 2015; Gilbert & Fiez, 2004; Pochon et al., 2002). Of particular importance, acute stress was shown to selectively diminish the contributions of model‐based learning strategies to behaviors through increased activation of the hypothalamic–pituitary–adrenal (HPA) axis (Otto, Raio, Chiang, Phelps, & Daw, 2013). Specifically, it was evidenced that acute stress exposure might impair prefrontal cortex functioning notably by increasing dopaminergic release, resulting therefore in enhanced habitual and automatic behaviors at the expense of flexible adaptive behaviors (for a review, see Arnsten, 2009). The ability to engage model‐based learning strategies requires cognitive resources to enable the implementation of controlled goal‐directed behaviors (Otto, Gershman, Markman, & Daw, 2013). A promising hypothesis linking stress sensitivity to motivated behaviors suggests that higher‐order cognitive functions might modulate the stress‐induced effect on the capacity to engage in goal‐directed actions. Converging with this idea, a study demonstrated that the detrimental effect of acute stress on the ability to engage model‐based strategies to guide behaviors was modulated by individual working memory capacity, with higher working memory capacity protecting against stress‐induced reduction in model‐based learning (Otto, Raio, et al., 2013).

So far, researchers have taken an active interest in investigating (a) the role of stress on reward responsiveness (Berghorst et al., 2013; Boecker et al., 2014; Bogdan & Pizzagalli, 2006; Ginty, 2013; Hanson et al., 2015; Porcelli et al., 2012), (b) the relationship between cognition and motivation (Botvinick et al., 2009; Satterthwaite et al., 2012; Vassena et al., 2014), and (c) the effect of stress on higher‐order cognitive functions (for a review, see Arnsten, 2009). Here, we used an event‐related fMRI task to test how unpredictable acute stressor (threat‐of‐shock) modulates reward responsiveness under variable levels of cognitive effort (working memory load) exerted for obtaining a monetary reward. Based on previous research, we hypothesized that the unpredictable acute stressor would increase striatal reactivity to cued reward during anticipation and would reduce striatal reactivity to reward during feedback delivery. According to the effort‐discounting effect, we expected that high working memory load would counteract the enhancing effect of stress on striatal reactivity to reward anticipation, but would strengthen the blunting effect of stress on the striatal reactivity to reward delivery. At the behavioral level, we hypothesized that both the unpredictable acute stressor and the higher cognitive load would reduce performance (as reflected by a slower reaction times and a decreased response accuracy), thus acting synergistically.

## 2 MATERIALS AND METHODS

### 2.1 Participants

Twenty‐three healthy, right‐handed adults (14 women, mean age: 24.7 ± 0.9, aged 20–37 years) participated in this study. Socioeconomic status was average relative to the Swiss population according to the index for individual socioeconomic level (IPSE; Genoud, 2011) (mean IPSE: 57.9 ± 3.4). Participants reported no current or past psychopathology, as well as no use of psychoactive drugs, as assessed by the Mini‐International Neuropsychiatric Interview (M.I.N.I.; Sheehan et al., 1998). In addition, no history of neurological or endocrine diseases was present among the sample.

### 2.2 General procedure

This study was approved by the local ethical review boards of Vaud and Fribourg region (Commission cantonale d'éthique de la recherche sur l'être humain [CER‐VD], study number 261/14) as well as of Bern region (Kantonale Ethikkommission Bern [KEK BE], study number 337/14) and all participants provided written informed consent. Before entering the scanner, the participants were trained on the task. During the fMRI scanning session, participants completed two blocks of the Fribourg reward task, one without (control condition) and one with the experimentally induced acute stressor (stress condition).

### 2.3 Fribourg reward task

**Figure 1:**
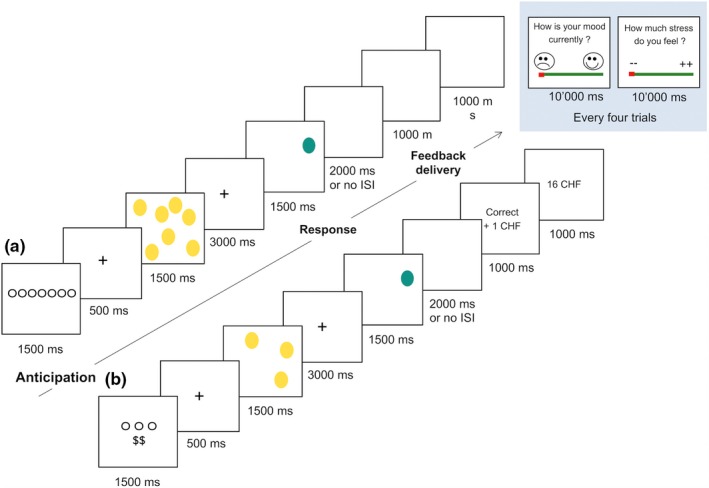
Illustration of (a) a not‐rewarded trial at the highest level of working memory (WM) load and (b) a rewarded trial at the easiest WM load of the Fribourg reward task

This event‐related fMRI task was adapted from the reward task developed by Martin‐Soelch et al. (2009) to elicit brain responses to reward anticipation and delivery. At the onset of each trial, a visual cue (1,500 ms) was presented informing participants of the effort level of working memory to expend (low, high) and the monetary reward associated with performance (“blank screen” for not‐rewarded trials or “$$” for rewarded trials). After the presentation of a fixation cross (500 ms), participants saw an array of yellow circles (3 or 7 circles, 1,500 ms). A fixation cross (3,000 ms) was presented before the visual target (1,500 ms). The visual target (a green circle) was displayed at any position on the screen and signaled that the participant should decide as quickly as possible whether this circle was at the same position as one of the circles presented previously. After response execution and a variable jittered interstimulus interval (ISI; 0 ms or 2,000 ms), the feedback screen (1,000 ms) informed the win (“blank screen” for not‐rewarded trials; “1 CHF” for rewarded trials) and was followed by a last screen (1,000 ms) indicating the cumulated amount of earned money (rewarded trials) or a blank screen (not‐rewarded trials). Every four trials, participants rated their mood and stress levels for a maximal duration of 20 s. Correct responses were associated with monetary gains (1 CHF) in the rewarded condition. Correct responses were not associated with monetary gains (0 CHF) in the not‐rewarded condition. All functional images were acquired within two distinct blocks. In the first one (i.e., control condition), no stressor was included during the task. In the second one (i.e., stress condition), a moderate stress was introduced through the administration of six unpredictable mild electric shocks to investigate its impact on reward responsiveness. In this task, the cognitive effort to expend was modulated with two levels of working memory load (low and high) corresponding to the number of circles to be remembered. Participants were informed that they would receive the total sum in cash at the end of the scanning session. Figure 1 details the timing of the events of a rewarded and a not‐rewarded trial.

### 2.4 Acute experimental stress manipulation

Participants were told that they may receive electrical shocks at any time during the second block of the experimental task (i.e., stress condition), while they were informed that no electrical shocks would be delivered during the first block. Six unpredictable mild electric shocks were delivered during the stress condition. Shocks were given on the external side of the nondominant left hand of participants via 6‐mm Ag/AgCl electrodes, using the SHK module of the Psychlab system (Contact Precision Instruments). The electrode wires were connected to a nonferromagnetic shock box placed on a table just beside the scanner. Before entering the scanner, a standard shock workup procedure was conducted to determine individual shock intensity (*M* = 1.07 mA ± 0.09), starting at the lowest level and increasing the intensity until the participant identified an “aversive, but not painful” feeling (Robinson et al., 2011). Highest allowable intensity level of the shock was 5 mA (milliamperes).

### 2.5 Self‐reported ratings of the experimental stressor manipulation

Every four trials of the event‐related Fribourg reward task, self‐reported ratings of mood and stress were assessed at the end of the trial using a Visual Analog Mood Scale (scaled from 0 to 9) adapted from Nyenhuis, Stern, Yamamoto, Luchetta, and Arruda (1997). For each participant, self‐reported ratings were averaged separately during the control condition and the stress condition and were entered into SPSS (Version 25.0; IBM SPSS Statistics).

### 2.6 MR data acquisition

Magnetic resonance imagery (MRI) acquisition was performed at the Department of Diagnostic and Interventional Neuroradiology of the University Hospital of Bern, Switzerland. The functional MRI images were acquired using a Siemens TrioTim syngo 3.0‐Tesla whole‐body scanner equipped with a 32‐channel head coil. MRI acquisition included 3D T1‐weighted (Magnetization Prepared Rapid Acquisition Gradient Echo; MPRAGE) images with the following settings: sagittal slices: 176; FOV: 256 mm × 256 mm; matrix size: 256 × 256; voxel size: 1.0 × 1.0 × 1.0 mm^3^; TR: 2,300 ms; TE: 2.32 ms; flip angle: 8°. During the event‐related task‐based fMRI, an echo‐planar imaging (EPI) pulse sequence was used with following settings: interleaved ascending slices: 38; FOV: 192 × 192 mm; matrix size: 64 × 64; voxel size: 3.0 × 3.0 × 4.0 mm^3^; TR: 2,000 ms; TE: 30 ms; flip angle: 90°. The event‐related task‐based fMRI included two blocks within one scanning session. Each block lasted on average 20 min. Stimuli were presented via goggles (VisualStimDigital MR‐compatible video goggles; Resonance Technology Inc.) with a visual angle of 60°, a resolution of 800 × 600 pixels, and 60 Hz refresh rate. The task was run using E‐Prime (version 2.0.10.353; Psychology Software Tools, Inc.). Total time in the scanner was approximately 60 min.

### 2.7 Analyses of working memory performance

A 2 × 2 × 2 repeated‐measures ANOVA with *Reward* (rewarded, not‐rewarded) × *Stress* (stress, control) × *Load* (low, high) as within‐subject factors was run using SPSS (version 25.0; IBM SPSS Statistics) on reaction times and response accuracy on the working memory task.

### 2.8 Analyses of the acute experimental stressor effect on self‐reported ratings

The effect of acute experimental stressor manipulation on self‐reported measurements of stress and mood was tested by computing the difference between self‐reported ratings during the control condition and the stress condition. A Wilcoxon signed‐rank test was applied using SPSS (version 25.0; IBM SPSS Statistics). A nonparametric test was used because of the non‐normally distributed mood ratings and two outliers among the stress ratings.

### 2.9 fMRI data analysis

#### 2.9.1 fMRI data preprocessing

All images were processed using Analysis of Functional NeuroImages (AFNI; Cox, 1996). Subjects with gross motion exceeding 3 mm were excluded from further analysis (averaged motion: 0.05 ± 0.01). The EPI images were preprocessed according to the following steps using *afni_proc.py*. Motion parameters from each block were used as separate regressors and did not differ significantly between the control condition (mean TR censored: 0.45%) and the stress condition (mean TR censored: 0.47%), *t*_(22)_ = −0.09, *p* > .05. To correct for motion, any EPI volume with an Euclidean mean of 0.3 mm shift from its preceding volume was censored from regression along with its preceding volume. Subject‐level exclusion for motion was based on the 0.3 mm censoring. In addition, TRs with more than 10% of (motion‐based) voxel outliers were censored. Subjects with more than 10% censored TRs were excluded from analysis. Three subjects were excluded based on these criteria, leaving a sample of *n* = 23. T1 images were first processed with FreeSurfer version 6.0.0 (Fischl, 2004) to obtain segmentation masks corresponding to the skull‐stripped brain, white matter, and ventricles. Whole‐brain masks were warped with standard normalization to Montreal Neurological Institute (MNI) space using the ICBM 2009a Nonlinear Symmetric atlas (Fonov, Evans, McKinstry, Almli, & Collins, 2009), and spatially smoothed with an isotropic 6 mm full‐width half maximum Gaussian kernel. Binary masks were averaged and thresholded at 0.95 (i.e., 95% overlap) to create a group‐level gray matter mask (Torrisi et al., 2018).

#### 2.9.2 fMRI data analysis

Statistical analysis was performed within the framework of the general linear model, as implemented in the AFNI program *3dDeconvolve*. Analyses focused on changes in BOLD contrast that occurred during reward anticipation and feedback delivery. To determine the effects of monetary reward, experimental stressor, and working memory load on BOLD responses, a general linear model was performed with stress (stress vs. control), reward (rewarded vs. not‐rewarded), and load (high vs. low) as fixed factors, and subjects as a random factor. To test a priori hypotheses focusing on the interaction effect between stress and working memory load on striatal sensitivity to reward during reward anticipation and feedback delivery, three regions of interest (ROIs) were created using the maximum probability atlas of Desai DKD maps in FreeSurfer (Desikan et al., 2006; Destrieux, Fischl, Dale, & Halgren, 2010; Fischl, 2004). ROIs included the bilateral NAcc, caudate nucleus, and putamen. Activation of voxels (i.e., parameter estimates) was averaged and extracted from each ROI mask in each condition and in each subject. Next, parameter estimates extracted from each ROI were entered and analyzed into SPSS. A 2 × 2 × 2 repeated‐measures ANOVA with reward (rewarded, not‐rewarded) × stress (stress, control) × load (low, high) as within‐subject factors was calculated for testing our hypotheses on striatal ROIs. Parameter estimates extracted from each ROI were normally distributed and satisfied the homogeneity of variance assumption. ROI activation analyses were corrected for multiple comparisons by applying a Bonferroni correction (*p*‐value = 0.05/3 = .017). A whole‐brain 2 × 2 × 2 repeated‐measures ANOVA was also conducted. To address the concerns of inflated false‐positive rates identified by Eklund, Nichols, and Knutsson (2016), whole‐brain activation maps were corrected for multiple comparisons by using a cluster‐based approach by conducting 10,000 Monte Carlo simulations using the AFNI program *3dClustSim*, after smoothness of noise in the dataset itself and from the residuals had been estimated for each subject and then averaged over all subjects with *3dFWHMx*. The updated *3dClustSim* version includes a mixed autocorrelation function (ACF) that better models non‐Gaussian noise structure (Cox, Chen, Glen, Reynolds, & Taylor, 2017). fMRI data were then thresholded using a voxelwise *p*‐value threshold of *p* < .001, and a minimum cluster size of *k* = 18, which corresponds to a whole‐brain, cluster‐level alpha of *p* < .05.

## 3 RESULTS

### 3.1 Effect of acute experimental stressor on self‐reported ratings

**Figure 2:**
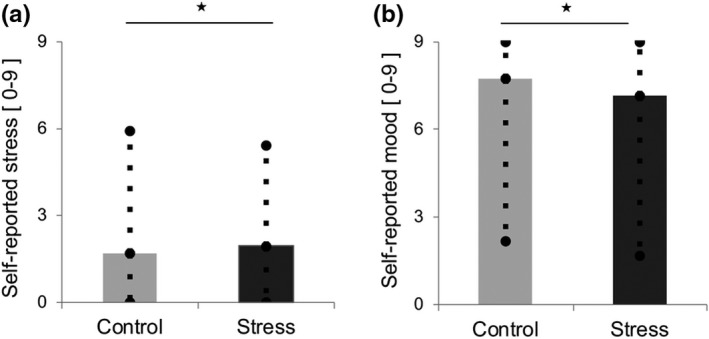
Effect of the stress condition on subjective stress and mood ratings during the Fribourg reward task. (a) Median and min./max. scores characterizing self‐reported stress in the control and stress conditions, scaled from 0 “not stressed at all” to 9 “very stressed.” (b) Median and min./max. scores characterizing self‐reported mood in the control and stress conditions, scaled from 0 “very negative mood” to 9 “very positive mood.” *p < .05

We first assessed whether self‐reported stress and negative mood ratings increased in the stress condition. A Wilcoxon signed‐rank test showed a significant increase in self‐reported stress in the stress condition (Mdn = 2.0; IR = 2.2) compared to the control condition (Mdn = 1.7; IR = 2.4), *Z* = −2.35, *p ≤ *.02. In addition, a significant decrease in the subjective mood ratings was induced by the stress condition (Mdn = 7.2; IR = 3.4) compared to the control condition (*Mdn* = 7.8; *IR *= 3.8), *Z* = −2.05, *p ≤ *.04 (Figure 2).

### 3.2 Working memory performance

#### 3.2.1 Response accuracy

**Figure 3:**
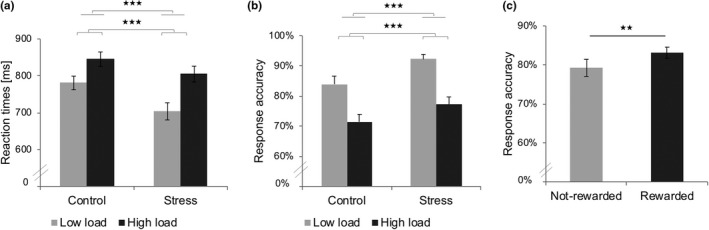
Working memory (WM) performance during the Fribourg reward task. (a) Averaged reaction times in the control versus stress conditions according to low and high WM load. (b) Averaged response accuracy in the control versus stress conditions according to low and high WM load. (c) Averaged response accuracy in rewarded and not‐rewarded trials. **p < .01, ***p < .001

As predicted, the repeated‐measures ANOVA on the response accuracy revealed a main effect of reward with significant increased response accuracy in rewarded trials (*M* = 83.1%; *SE* = 1.4%) compared to not‐rewarded trials (*M* = 79.2%; *SE* = 2.2%), *F*_1,22_ = 9.2, *p ≤ *.006, *η* ^2^ = 0.29. In accordance with our expectation, a main effect of working memory load showed a significant decreased response accuracy in trials under high working memory load (*M* = 74.3%; *SE* = 2.1%) compared to low working memory load (*M* = 88.0%; *SE* = 1.8%), *F*_1,22_ = 55.0, *p <* .001, *η* ^2^ = 0.71. Unexpectedly, a main effect of stress appeared with increased response accuracy in the stress condition (*M* = 84.7%; *SE* = 1.8%) compared to the control condition (*M* = 77.7%; *SE* = 2.1%), *F*_(1,22) _= 13.4, *p ≤* .0.001, *η* ^2^ = 0.38 (Figure 3).

#### 3.2.2 Reaction times (RT)

Corroborating our expectation, the repeated‐measures ANOVA on the reaction times showed a significant main effect of working memory load indicating slower reaction times in trials under high load (*M* = 825.0 ms; *SE* = 18.1 ms) compared to low load (*M* = 742.1 ms; *SE* = 19.0 ms), *F*_1,22_ = 75.1, *p* < .001, *η* ^2^ = 0.77. The stress condition led to significant faster responses (*M* = 754.1 ms; *SE* = 21.4 ms) in comparison with reaction times in the control condition (*M* = 813.0 ms; *SE* = 17.0 ms), *F*_1,22_ = 16.9, *p* < .001, *η* ^2^ = 0.43. The effect of reward did not significantly affect reaction times (Figure 3).

### 3.3 fMRI results

#### 3.3.1 ROI analysis: Striatal activations during reward anticipation

**Figure 4:**
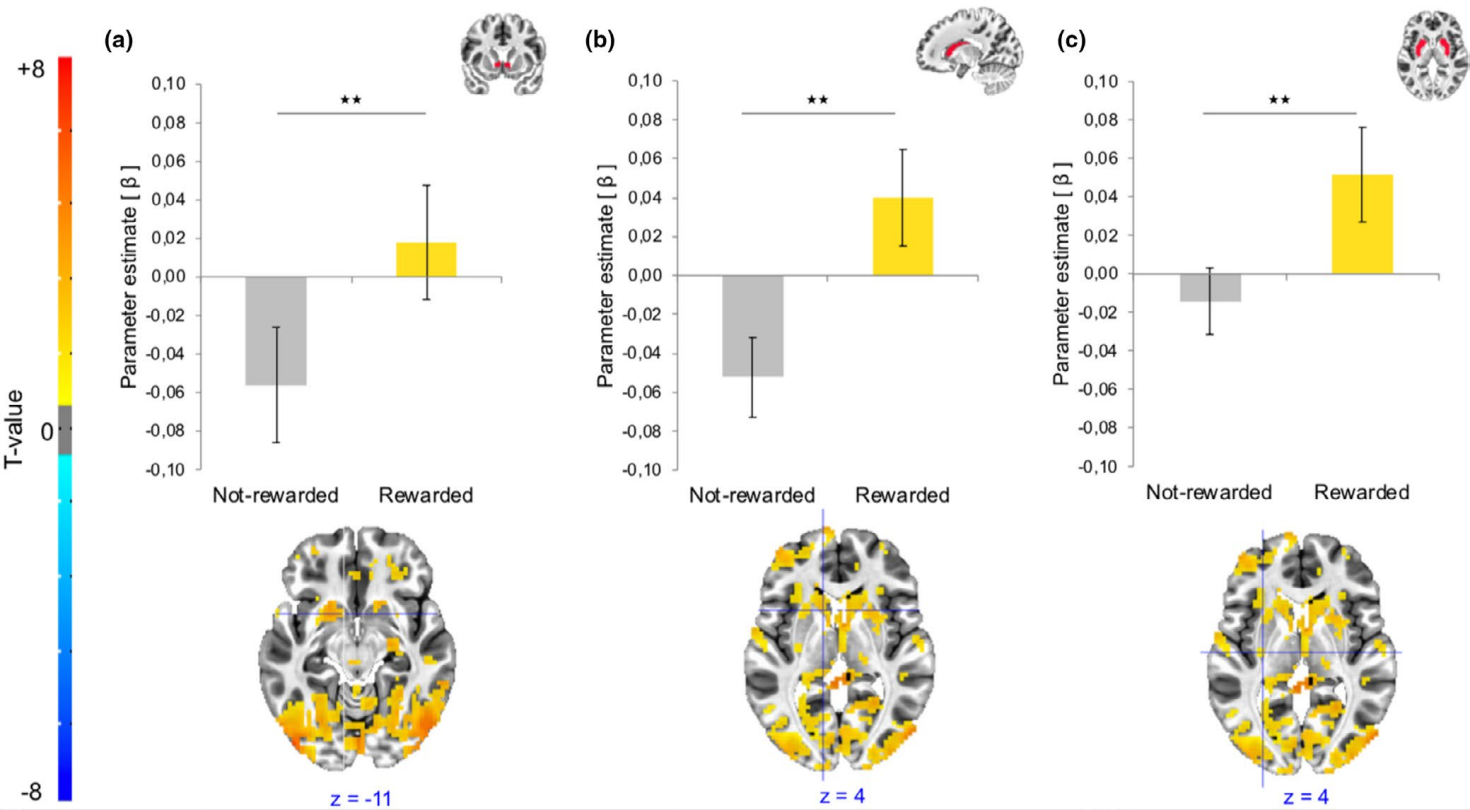
Illustration of the main effect of reward during the anticipation phase. Significant main effect of reward (rewarded vs. not‐rewarded) in the bilateral (a) nucleus accumbens, (b) caudate nucleus, and (c) putamen. Parameter estimates (βeta weights) mean with standard errors are presented at the top of the figure. Statistical parametric maps corresponding to the contrasts of interest during anticipation are presented below. Whole‐brain activations are corrected for multiple comparisons using a voxelwise *p*‐value threshold of *p* < .001, and a minimum cluster size of *k* = 18, which corresponds to a whole‐brain, cluster‐level alpha of *p* < .05. A voxelwise *p*‐value threshold of *p* < .05 was used here for visualization purpose. **p < .01

The anticipation of potential monetary rewards induced a significant main effect of reward with increased activation in the NAcc (*F*_1,22_ = 9.60, *p* ≤ .006, *η* ^2^ = 0.30, Bonferroni‐corrected), caudate nucleus (*F*_1,22_ = 12.51, *p* ≤ .002, Bonferroni‐corrected), and putamen (*F*_1,22_ = 9.11, *η* ^2^ = 0.29, *p* ≤ .007, Bonferroni‐corrected) in rewarded trials compared to not‐rewarded trials. Both threat‐of‐shock and level of working memory load did not show any significant effect on the neural correlates of reward anticipation (Figure 4). Main and interaction effects in each condition from each ROI mask are detailed in the appendix (Table A1).

#### 3.3.2 ROI analysis: Striatal activations during feedback delivery

**Figure 5:**
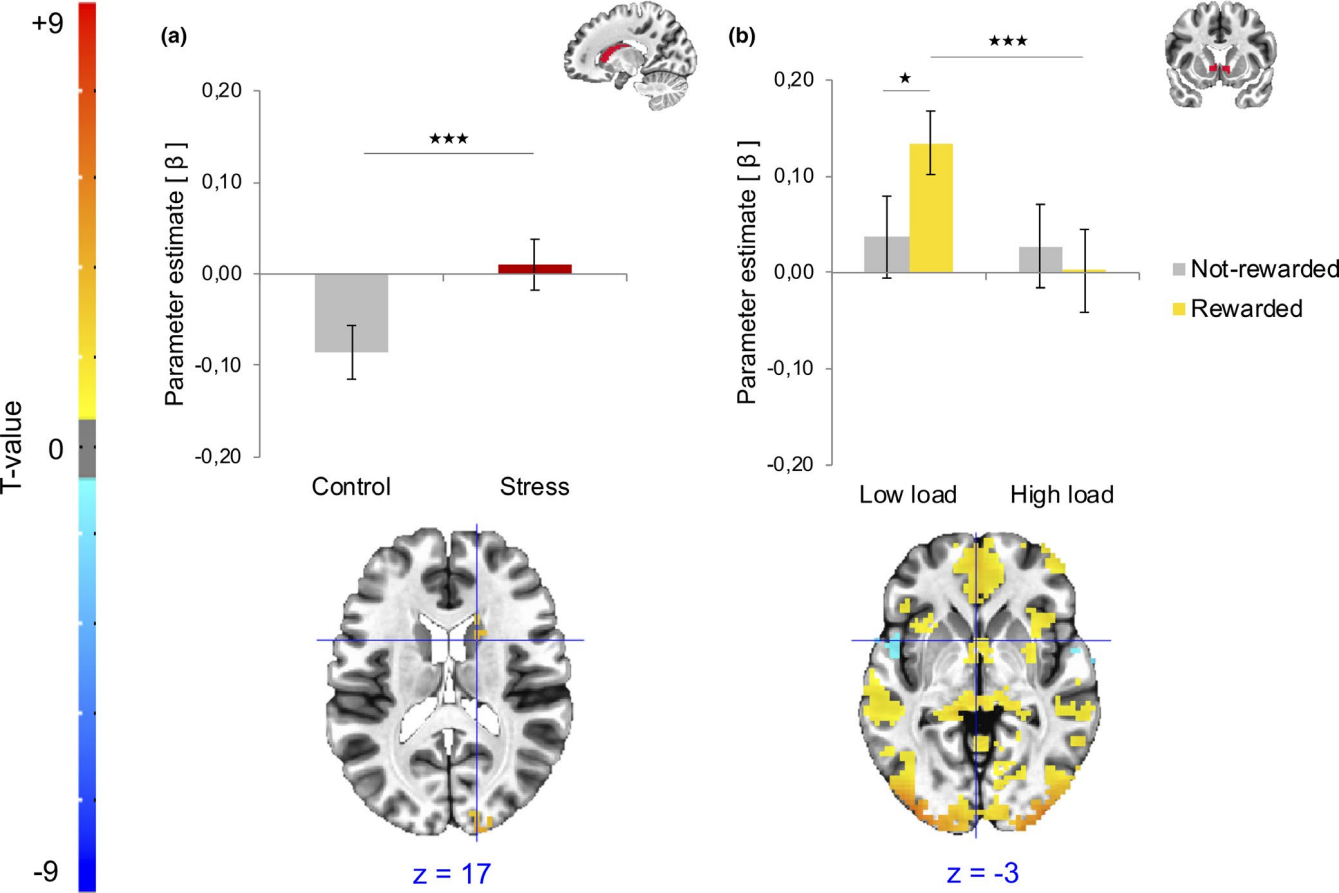
Statistical parametric maps during feedback delivery showing (a) a main effect of stress in the bilateral caudate nucleus with significant increased activation in the stress condition compared to the control condition. (b) Reward by working memory (WM) load interaction in the nucleus accumbens, with significant decreased responsiveness to reward delivery under high compared to low WM load. Whole‐brain activations are corrected for multiple comparisons using a voxelwise *p*‐value threshold of *p* < .001, and a minimum cluster size of *k* = 18, which corresponds to a whole‐brain, cluster‐level alpha of *p* < .05. A voxelwise *p*‐value threshold of *p* < .05 was used here for visualization purpose. *p < .05, ***p < .001

During feedback delivery, a main effect of stress was present in the caudate nucleus with higher activation in the stress condition compared to the control condition (*F*_1,22_ = 6.81, *p* ≤ .016, *η* ^2^ = 0.24, Bonferroni‐corrected). Additionally, a significant reward by working memory load interaction occurred in the NAcc (*F*_1,22_ = 7.76, *p* ≤ .011, *η* ^2^ = 0.26, Bonferroni‐corrected). Post hoc analysis indicated that the NAcc responses to reward delivery depended on the level of working memory load, with greater responsiveness to reward delivery in low working memory load compared to high working memory load (*t*_22_ = 3.85, *p* < .001, Bonferroni‐corrected; Figure 5). Main and interaction effects in each condition from each ROI mask are detailed in the appendix (Table A1).

Significant whole‐brain clusters in contrasts discussed above are presented in Table 1 (whole‐brain corrected using a cluster‐level alpha of *p* < .05; see Tables A2 and A3 in the appendix for a comprehensive report of whole‐brain activations in all conditions).

**Table 1:** Significant whole‐brain clusters (cluster size corrected) for (a) the main effect of reward (rewarded vs. not‐rewarded) during the anticipation phase, and (b) the main effect of stress (stress vs. control), as well as interaction effect between reward (rewarded vs. not‐rewarded) and working memory (WM) load (high vs. low) during the feedback delivery phase

Activated clusters in brain regions	Side	MNI coordinates (LPI)			Cluster size	*T‐*Value
		*x*	*y*	*z*		
1. Anticipation						
Main effect of reward: rewarded > not‐rewarded trials						
Lateral occipital	L	−47	−86	−11	456	4.32
Fusiform	R	50	−65	−20	297	5.56
Superior parietal	L	−8	−80	53	70	4.03
Lateral occipital	R	38	−92	14	61	4.11
Superior parietal	R	29	−59	68	34	4.13
Supramarginal	L	−53	−38	56	31	4.45
Superior parietal	R	32	−41	50	30	4.63
Rostral middle frontal	L	−41	50	2	27	4.60
Superior parietal	L	−20	−83	41	27	4.75
Lingual	R	8	−83	−17	25	3.84
Cerebral white matter	L	−20	−71	8	24	4.89
Superior parietal	R	23	−83	50	21	5.68
2. Feedback delivery						
Main effect of stress: stress > control conditions						
Superior parietal	R	20	−92	38	42	5.11
Superior frontal	L	−2	11	38	31	4.69
Lateral occipital	R	17	−101	20	28	4.72
Insula	L	−38	−23	5	22	5.00
PCC	R	11	−26	41	20	5.11
Caudate	R	17	8	17	18	3.97
Postcentral	L	−56	−26	47	18	3.99
Interaction effect Reward × WM load: rewarded > not‐rewarded trials in the low load condition						
Lateral occipital	L	−47	−86	−11	1,867	4.95
Superior frontal	L	−2	62	2	247	4.36
Superior parietal	L	−32	−65	56	126	3.98
PCC	R	2	−29	32	78	4.80
Superior temporal	L	−62	−35	5	74	8.13
Inferior parietal	R	44	−59	59	47	3.88
Precentral	L	−47	5	38	41	4.00
Superior parietal	R	44	−47	56	41	4.38
Insula	R	32	14	−20	26	4.08
Superior frontal	L	−2	38	23	26	4.18
Cerebellum	L	−29	−74	−47	24	4.46

Note

Whole‐brain activations presented for every specific contrast are corrected for multiple comparisons using a cluster‐based approach with a voxelwise *p*‐value threshold of *p* < .001 and a minimum cluster size of *k* = 18, which corresponds to a cluster‐level alpha of *p* < .05. LPI means that *x* increases from left to right, *y* increases from posterior to anterior, *z* increases from inferior to superior.

Abbreviations: L, left; R, right.

## 4 DISCUSSION

The aim of the current study was to investigate the effects of an acute stressor induced experimentally by threat‐of‐shock and of cognitive effort (high vs. low working memory load) on the striatal responsiveness to monetary reward, during reward anticipation and feedback notification. To the best of our knowledge, this is the first study specifically exploring how stress induction and working memory load modulate neural reactivity to reward during the anticipation and delivery phases. Consistent with prior fMRI studies, stress manipulation successfully induced a negative mood and increased self‐reported stress in participants (Bogdan & Pizzagalli, 2006; Grillon, Ameli, Foot, & Davis, 1993). Contrary to our expectations, no significant interaction occurred among stress, cognitive load, and reward during the anticipation of potential monetary rewards. Enhanced striatal reactivity to potential reward occurred in rewarded trials, irrespective of the modulation by the experimental stressor or by the cognitive effort to expend for getting the reward. Crucially, both stress and cognitive effort affected striatal activation during feedback delivery, but these factors did not interact to modulate reward responsiveness. First, striatal reactivity to reward delivery was modulated by the level of working memory effort that was expended to obtain the reward, with significantly decreased responsiveness to monetary reward in the ventral striatum following high, compared to low, cognitive effort. Second, stress strengthened reactivity in the dorsal striatum during feedback delivery and enhanced cognitive performance.

The present study indicates that both ventral and dorsal striatum responded to potential monetary reward during the cue‐triggered anticipation irrespective of the presence of an experimental stressor or of the level of cognitive effort engaged for obtaining the reward. These findings converge with previous data demonstrating increased activation in striatal regions in response to anticipated monetary rewards (Knutson & Greer, 2008; Miller, Shankar, Knutson, & McClure, 2014; Rademacher et al., 2013). Significant increase in striatal responsiveness to anticipated rewards in our study was additionally consistent with enhanced behavioral performance in rewarded trials, compared to not‐rewarded trials. Collectively, our results showed that potential reward improved response accuracy and decreased reaction times. These behavioral results are in accordance with findings pointing out that reward was able to increase cognitive performance (Choi, Padmala, & Pessoa, 2015; Savine, Beck, Edwards, Chiew, & Braver, 2010), as evidenced, for instance, in a spatial working memory task (Kennerley & Wallis, 2009). Increased striatal responsiveness to anticipated reward and improved behavioral performance might reveal enhanced incentive‐triggered motivation. In contrast to our hypotheses and recent studies indicating that stress (Kumar et al., 2014) and greater cognitive demands (Vassena et al., 2014) led to higher involvement of the neural circuits underlying motivated behaviors, no effect of the experimental stressor together with the level of cognitive load modulated the neural reactivity to reward.

During feedback delivery, the striatal responsiveness to reward delivery was modulated by the level of cognitive effort deployed for obtaining the reward. Specifically, reward responsiveness in the ventral striatum decreased following high, compared to low, cognitive effort. Our findings converge with mounting evidence demonstrating that a higher amount of both physical (e.g., Apps, Grima, Manohar, & Husain, 2015; Bonnelle et al., 2015; Kurniawan et al., 2010) and cognitive (e.g., Botvinick et al., 2009; Krigolson, Hassall, Satel, & Klein, 2015; Stoppel et al., 2011) efforts diminish the value attached to a reward. Also, data showing decreased NAcc responsiveness during reward delivery following the exertion of higher cognitive effort support directly the present results (Botvinick et al., 2009). In line with the idea that the value attributed to a potential reward is inversely related to the degree of effort required for obtaining it (Botvinick et al., 2009), our findings suggest that the magnitude of cognitive effort exerted had a discounting effect on reward value, reflected by decreased striatal responsiveness to reward delivery. While dopaminergic neurotransmission has been strongly involved in the willingness and in the ability to expend higher effort for getting a reward (Boehler et al., 2011; Treadway et al., 2012; Wardle, Treadway, Mayo, Zald, & de Wit, 2011), a possible hypothesis explaining the effort‐discounting effect which occurred during reward delivery is that effort expenditure might have engaged the same dopaminergic corticolimbic brain network as the one involved during the attribution of reward value, both competing for the same cognitive resources (Stoppel et al., 2011; Vassena et al., 2014).

Interestingly, the acute experimental stressor strengthened activation in the caudate nucleus during feedback delivery, irrespective of the level of cognitive effort or of the presence of incentive. Increased threat‐related recruitment of the caudate nucleus might be due to heightened arousal mediated by increased dopamine release in the striatum, as previously suggested in the NAcc (Cabib & Puglisi‐Allegra, 2012; Pruessner, Champagne, Meaney, & Dagher, 2004; Soares‐Cunha, Coimbra, Sousa, & Rodrigues, 2016). In humans, enhanced dopamine signaling in the striatum has been linked with the arousing effect of novel or alerting cues (Horvitz, 2002; Soares‐Cunha et al., 2016) and with the attentional capture by salient cues (Anderson, 2017). Together with the caudate nucleus, the superior frontal regions, the superior parietal lobule, and the anterior insula also showed increased threat‐related activation. This finding is in line with a recent study evidencing enhanced recruitment of the caudate nucleus, the anterior insula, and regions of the frontoparietal attention network under threat‐of‐shock (Torrisi et al., 2016). In particular, stronger recruitment of superior frontal regions during stress exposure in our task converge with data showing that acute stress exposure might strengthen cognitive arousal mediated possibly by increased dopaminergic neurotransmission in prefrontal regions, resulting in higher working memory performance (Arnsten & Jin, 2014; Weerda, Muehlhan, Wolf, & Thiel, 2010). Accordingly, enhanced threat‐related activation in prefrontal and parietal regions was paralleled by improved cognitive performance under threat‐of‐shock in our study. Indeed, stress elicited higher response accuracy and faster reaction times. Since our study did not manipulate dopamine pharmacologically, interpretations on the potential involvement of the dopamine system should be considered with caution. Nevertheless, these findings converge with behavioral data in animals (Yuen et al., 2011) and humans (Duncko & Johnson, 2009; Torrisi et al., 2016), showing threat‐related enhanced working memory performance (Duncko & Johnson, 2009). Altogether, the present findings suggest that unpredictable stress exposure might contribute to the dysregulation of cognitive and emotional arousal, resulting consequently in a sensitization of the dorsal striatum reactivity to outcomes generally. Also, the present results indicate that the ability to encode reward value is modulated by effort expenditure, with a propensity to depreciate reward value following high‐demanding cognitive effort.

This study comes with some limitations deserving mention. First, given our within‐subjects design and that both blocks with and without stressor took place on the same day, no randomization was possible between blocks, in order to avoid the potential bleeding of negative effects induced by threat‐of‐shock into the control condition. However, this methodology permits to avoid the methodological issues of scanning in different days. Second, although stress manipulation successfully induced negative affect and strengthened self‐reported stress, no physiological data are supporting the effectiveness of the stress manipulation. Third, the potential temporal autocorrelation of first‐level imaging data is a limitation that should be taken into account. A final limitation is that the sample size was relatively small, and thus, the results should be considered preliminary, in need of replication.

In conclusion, the present study provides initial evidence that both acute stressor and cognitive load modulate neural responsiveness during feedback delivery but not during the anticipation of potential monetary reward. Our results indicate that reward value decreases under demanding cognitive load. High cognitive effort might represent a cost, which decreases the value of the reward, and shifts attention away from the reward. Of particular relevance, threat‐of‐shock facilitates behavioral performance, probably by increasing arousal and attentional focus through the recruitment of striatal regions and areas involved in the frontoparietal attention network (Balderston et al., 2017; McEwen & Sapolsky, 1995; Torrisi et al., 2016). In line with a recent meta‐analytic study showing striatal hyperactivation during reward notification in individuals with substance addiction (Luijten, Schellekens, Kühn, Machielse, & Sescousse, 2017), these findings extend previous work by suggesting that sustained stress exposure might go along with dysregulated arousal, resulting possibly in increased risk for the development of maladaptive incentive‐triggered motivation. In sum, this study brings new insight that might help to build a framework to understand common stress‐related disorders involving disturbances of the reward system, cognitive deficits, and abnormal stress reactivity.

## CONFLICT OF INTEREST

We certify that none of the authors has a financial interest to report.

## Data Availability

The data that support the findings of this study are available from the corresponding author upon reasonable request.

## APPENDIX 1

**Table A1:** Main and interaction effects of within‐subject contrasts in the bilateral nucleus accumbens (NAcc), caudate nucleus, and putamen

Within‐subjects contrasts	Stress	Reward	WM load	Anticipation									Delivery								
				NAcc			Caudate nucleus			Putamen			NAcc			Caudate nucleus			Putamen		
				*F*_1,22_	*p*	*η* ^2^	*F*_1,22_	*p*	*η* ^2^	*F*_1,22_	*p*	*η* ^2^	*F*_1,22_	*p*	*η* ^2^	*F*_1,22_	*p*	*η* ^2^	*F*_1,22_	*p*	*η* ^2^
Stress	Stress versus Control			0.33	.57	0.02	0.27	.61	0.01	0.22	.64	0.01	0.75	.40	0.03	**6.81**	**.016**	**0.24**	6.08	.02	0.22
Reward		R versus NR		**9.60**	**.01**	**0.30**	**12.51**	**.00**	**0.36**	**9.11**	**.01**	**0.29**	0.05	.83	0.00	1.17	.29	0.05	0.02	.88	0.88
Load			High versus Low	0.37	.55	0.02	3.13	.09	0.13	4.44	.05	0.17	6.35	.02	0.33	6.20	.02	0.22	0.83	.37	0.37
Stress × Reward	Stress versus Control	R versus NR		0.00	.95	0.00	0.27	.61	0.01	5.03	.04	0.19	0.30	.59	0.01	0.04	.85	0.00	0.17	.69	0.69
Stress × Load	Stress versus Control		High versus Low	0.72	.41	0.03	0.04	.85	0.00	0.09	.76	0.00	0.00	.99	0.00	1.22	.28	0.05	1.04	.32	0.32
Reward × Load		R versus NR	High versus Low	1.05	.32	0.05	0.03	.87	0.00	0.46	.50	0.02	**7.76**	**.01**	**0.26**	5.10	.03	0.19	2.34	.14	0.14
Stress × Reward × Load	Stress versus Control	R versus NR	High versus Low	0.00	.99	0.00	0.90	.36	0.04	4.47	.05	0.17	0.15	.70	0.01	0.40	.53	0.02	0.17	.68	0.69

Note

Analyses of region‐of‐interest activations were corrected for multiple comparisons by applying a Bonferroni correction (*p*‐value* < *.017). Partial eta squared (*η* ^2^) represents the proportion of total variance accounted for by the factor, while excluding other factors from the total explained variance (i.e., nonerror variation) in the repeated‐measures ANOVA (Pierce, Block, & Aguinis, 2004). Partial eta squared (*η* ^2^) values range from 0 to 1. Values highlighted in bold refer to significant results at *p* < .05.

Abbreviations: *F*, *F*‐statistic with degrees of freedom for effect and error; NR, not‐rewarded; R, rewarded; WM, working memory; *η* ^2^, partial eta squared.

**Table A2:** Significant whole‐brain clusters (cluster size corrected) for the main effects of stress, reward, and working memory (WM) load, as well as their interactions during the anticipation phase

Activated clusters in brain regions	Side	MNI coordinates (LPI)			Cluster size	*T‐*Value
		*x*	*y*	*z*		
Main effect of reward: rewarded > not‐rewarded trials						
Lateral occipital	L	−47	−86	−11	456	4.32
Fusiform	R	50	−65	−20	297	5.56
Superior parietal	L	−8	−80	53	70	4.03
Lateral occipital	R	38	−92	14	61	4.11
Superior parietal	R	29	−59	68	34	4.13
Supramarginal	L	−53	−38	56	31	4.45
Superior parietal	R	32	−41	50	30	4.63
Rostral middle frontal	L	−41	50	2	27	4.60
Superior parietal	L	−20	−83	41	27	4.75
Lingual	R	8	−83	−17	25	3.84
Cerebral white matter	L	−20	−71	8	24	4.89
Superior parietal	R	23	−83	50	21	5.68
Main effect of WM load: high > low loads						
Lingual	L	−1	−85	0	1675	7.83
Interaction effect: Reward × Stress						
Rewarded > not‐rewarded trials in the control condition						
Inferior temporal	L	−50	−62	−20	203	3.81
Lateral occipital	R	44	−77	−17	126	5.01
Superior parietal	L	−8	−77	53	120	4.32
Lateral occipital	L	−29	−98	14	62	5.42
Superior parietal	R	23	−80	50	33	3.93
Lateral occipital	R	32	−95	20	21	4.27
Rostral middle frontal	L	−35	53	−2	20	4.42
Superior parietal	L	−35	−62	53	18	5.11
Rewarded > not‐rewarded trials in the stress condition						
Lateral occipital	L	−47	−86	−11	210	4.96
Cerebellum	R	50	−71	−26	25	4.03
Lateral occipital	R	53	−74	−8	22	4.35
Parietal occipital	L	−17	−71	11	18	4.05
Postcentral	R	35	−35	47	18	4.29
Interaction effect: Reward × WM load						
Rewarded > not‐rewarded trials in the low load condition						
Lateral occipital	L	−44	−86	−11	227	6.14
Fusiform	R	50	−65	−20	152	4.12
Lateral occipital	R	38	−92	14	64	4.33
Inferior parietal	L	−32	−77	29	29	4.50
Superior parietal	L	−44	−47	56	21	3.80
Sulcus parieto‐occipital	L	−20	−71	11	18	4.38
Superior parietal	L	−23	−65	53	18	4.96
Rewarded > not‐rewarded trials in the high load condition						
Fusiform	L	−47	−62	−20	211	4.40
Lateral occipital	R	50	−77	−14	61	5.62
Superior parietal	L	−29	−68	53	30	4.41
Lingual	R	2	−80	−5	27	4.50
Lateral occipital	L	−29	−98	14	18	4.20
Interaction effect: Stress × WM load						
High > low loads in the control condition						
Lingual	R	20	−77	−14	1,214	6.20
High > low loads in the stress condition						
Cerebellum	R	20	−80	−14	856	7.05
Interaction effect: Reward × Stress ×WM load						
Rewarded > not‐rewarded trials in the low load and control conditions						
Lateral occipital	L	−41	−83	−14	34	4.16
Inferior parietal	L	−38	−92	14	30	3.99
Fusiform	R	47	−65	−20	20	4.20
Rewarded > not‐rewarded trials in the high load and control conditions						
Fusiform	L	−43	−67	−15	99	4.61
Lateral occipital	R	46	−74	−14	24	4.16
Superior parietal	L	−13	−73	48	24	5.12
Lateral occipital	L	−31	−93	15	21	5.02
Rewarded > not‐rewarded trials in the low load and stress conditions						
Lateral occipital	L	−47	−86	−11	63	3.84
Lateral occipital	L	−26	−98	14	45	4.23
Lateral occipital	R	38	−77	−11	25	4.52
Supramarginal	L	−50	−32	50	21	4.41
Rewarded > not‐rewarded trials in the high load and stress conditions						
Lateral occipital	L	−50	−83	−5	36	5.15
Fusiform	L	−35	−59	−17	25	4.66
Fusiform	L	−35	−74	−17	17	4.29
Cerebellum	L	−11	−68	−17	17	7.71

Note

Whole‐brain activations presented for every specific contrast are corrected for multiple comparisons using a cluster‐based approach with a voxelwise *p*‐value threshold of *p* < .001 and a minimum cluster size of *k* = 18, which corresponds to a cluster‐level alpha of *p* < .05. LPI means that *x* increases from left to right, *y* increases from posterior to anterior, *z* increases from inferior to superior.

Abbreviations: L, left; R, right.

**Table A3:** Significant whole‐brain clusters (cluster size corrected) for the main effects of stress, reward, and working memory (WM) load, as well as their interactions during the feedback delivery phase

Activated clusters in brain regions	Side	MNI coordinates (LPI)			Cluster size	*T‐*Value
		*x*	*y*	*z*		
Main effect of reward: rewarded > not‐rewarded trials						
Lateral occipital	L	−47	−86	−11	2,859	5.38
Rostral ACC	R	2	47	8	626	6.57
Superior parietal	R	35	−68	59	157	4.30
Lateral orbitofrontal	R	47	26	−17	78	5.58
Middle temporal	L	−65	−38	5	72	5.37
Superior parietal	L	−29	−68	44	72	4.62
Superior temporal	L	−50	23	−11	52	4.10
Cerebellum	L	−5	−56	−35	48	5.70
Rostral middle frontal	R	50	44	23	41	4.65
Thalamus	L	−2	−2	8	28	3.82
Precuneus	L	−11	−62	8	28	3.97
Precentral	L	−50	8	38	28	5.17
Lateral occipital	L	−11	−104	14	26	−5.40
Supramarginal	R	65	−29	35	22	−3.83
Ventral DC	L	−2	−14	−14	20	5.80
Rostral middle frontal	L	−50	38	20	18	5.51
Main effect of stress: stress > control conditions						
Superior parietal	R	20	−92	38	42	5.11
Superior frontal	L	−2	11	38	31	4.69
Lateral occipital	R	17	−101	20	28	4.72
Insula	L	−38	−23	5	22	5.00
PCC	R	11	−26	41	20	5.11
Caudate	R	17	8	17	18	3.97
Postcentral	L	−56	−26	47	18	3.99
Main effect of WM load: high > low loads						
Amygdala	L	−17	−2	−17	29	−4.24
Superior frontal	L	−2	68	−2	18	−4.18
Interaction effect: Reward × Stress						
Rewarded > not‐rewarded trials in the control condition						
Lateral occipital	L	−47	−86	−11	991	5.32
Lateral occipital	R	44	−89	−11	863	7.83
Superior frontal	R	2	53	2	379	7.15
Middle temporal	L	−59	−56	14	149	4.52
PCC	L	−2	−29	32	147	5.84
Hippocampus	L	−23	−20	−14	121	3.97
Ventral DC	R	20	−26	−8	83	5.36
Pars orbitalis	R	50	26	−14	52	4.06
Thalamus	L	−2	−2	8	38	4.30
Superior temporal	L	−47	23	−17	34	3.94
Cerebellum	R	2	−83	−38	28	3.95
Precuneus	R	8	−56	8	25	5.32
Inferior parietal	R	47	−50	56	21	4.74
Superior frontal	R	2	32	32	18	4.40
Rewarded > not‐rewarded trials in the stress condition						
Lateral occipital	L	−47	−86	−11	735	4.96
Lateral occipital	R	50	−68	−20	709	4.11
Superior frontal	R	2	50	11	243	5.99
Superior parietal	R	35	−65	59	82	4.15
PCC	R	2	−29	32	79	6.03
Superior parietal	L	−29	−68	47	72	5.89
Cerebellum	R	2	−83	−38	51	5.10
Cerebellum	L	−11	−56	−35	31	4.17
Inferior parietal	R	32	−74	41	25	3.83
Precentral	L	−47	5	38	22	3.81
Rostral middle central	R	50	44	23	20	4.12
Interaction effect: Reward × Load						
Rewarded > not‐rewarded trials in the low load condition						
Lateral occipital	L	−47	−86	−11	1867	4.95
Superior frontal	L	−2	62	2	247	4.36
Superior parietal	L	−32	−65	56	126	3.98
PCC	R	2	−29	32	78	4.80
Superior temporal	L	−62	−35	5	74	8.13
Inferior parietal	R	44	−59	59	47	3.88
Precentral	L	−47	5	38	41	4.00
Superior parietal	R	44	−47	56	41	4.38
Insula	R	32	14	−20	26	4.08
Superior frontal	L	−2	38	23	26	4.18
Cerebellum	L	−29	−74	−47	24	4.46
Rewarded > not‐rewarded trials in the high load condition						
Lateral occipital	L	−47	−86	−11	927	5.58
Lateral occipital	R	50	−68	−20	656	4.14
Rostral ACC	R	2	47	8	384	5.39
Isthmus cingulate	R	11	−53	5	110	4.57
Ventral DC	R	17	−29	−8	55	4.98
PCC	L	−2	−29	32	42	5.02
Cerebellum	R	2	−83	−38	30	4.52
Cerebellum	L	−5	−56	−35	26	5.21
Lateral orbitofrontal	R	47	23	−17	25	4.14
Rostral middle frontal	R	50	44	23	17	4.16
Interaction effect: Stress × WM load						
High > low loads in the control condition						
Superior parietal	R	38	−47	65	27	−3.90
High > low loads in the stress condition						
Superior frontal	L	−2	62	−2	50	−4.49
Amygdala	L	−17	−2	−17	31	−5.60
Hippocampus	R	23	−14	−14	19	−5.82
Interaction effect: Reward × Stress ×WM load						
Rewarded > not‐rewarded trials in the low load and control conditions						
Lateral occipital	L	−47	−86	−11	794	4.82
Lateral occipital	R	44	−89	−11	674	6.39
Superior frontal	R	2	53	2	129	5.75
Superior temporal	L	−62	−35	5	74	6.21
Insula	L	−29	17	−8	27	4.09

Note

Whole‐brain activations presented for every specific contrast are corrected for multiple comparisons using a cluster‐based approach with a voxelwise *p*‐value threshold of *p* < .001 and a minimum cluster size of *k* = 18, which corresponds to a cluster‐level alpha of *p* < .05. LPI means that *x* increases from left to right, *y* increases from posterior to anterior, *z* increases from inferior to superior.

Abbreviations: L, left; R, right.
